# Long-term primary culture of a clear cell ovarian carcinoma reveals an epithelial–mesenchymal cooperative interaction

**DOI:** 10.1186/s12935-015-0243-8

**Published:** 2015-09-24

**Authors:** Alicia A. Goyeneche, Michael Koch, Maria C. Bell, Carlos M. Telleria

**Affiliations:** Division of Basic Biomedical Sciences, Sanford School of Medicine, The University of South Dakota, Vermillion, SD USA; Department of Pathology, Sanford School of Medicine, The University of South Dakota, Sioux Falls, SD USA; Department of Obstetrics and Gynecology, Sanford School of Medicine, The University of South Dakota, Sioux Falls, SD USA; Department of Pathology, Faculty of Medicine, McGill University, 3775 University Street, Montreal, QC H3A 2B4 Canada

**Keywords:** Ovarian clear cell carcinoma, E-cadherin, Vimentin, Epithelial, Mesenchymal, Hepatocyte nuclear factor 1β

## Abstract

**Background:**

We studied a primary culture developed from a biopsy of a clear cell carcinoma of the ovary (O-CCC) by (a) assessing its capacity to retain in vitro pathological features of the tumor of origin; (b) characterizing the main cells released from the complex mass without forced purification of any particular cellular entity; and (c) investigating its long-term proliferative capacity.

**Methods:**

A primary cell culture was developed from a pelvic mass diagnosed as an O-CCC. The morphological analysis of the cell culture was carried out by phase contrast microscopy. Markers of epithelial, mesenchymal, and tumor initiating cells were evaluated by immunocytochemistry. Cell proliferation was studied by detection of bromodeoxyuridine (BrdU) incorporated into newly synthesized DNA. As a biomarker of O-CCC, we assessed the expression of hepatocyte nuclear factor (HNF) 1β.

**Results:**

We show that cells with epithelial morphological features express E-cadherin and expand with time in culture, a fact that the incorporation of BrdU confirms. Cells with mesenchymal-like characteristics that express the mesenchymal marker vimentin, however, allocate to the edges of the epithelial compartment. Moreover, we found that some cells with epithelial features also expressed vimentin. At the beginning of incubation, over 60 % of primary cells expressed the O-CCC marker HNF1β; such percentage declined upon passaging. We show that epithelial not mesenchymal cells undergo DNA replication, and that few cells in both epithelial and mesenchymal compartments express the stem-like tumor antigen CD133.

**Conclusions:**

We provide proof-of-principle that cells separated in bulk from a biopsy of an O-CCC can be maintained in culture for several months, and that two consistent cellular compartments—one epithelial that retains the O-CCC marker HNF1β, and another mesenchymal—persist, and seem to have a cooperative interaction leading to the multiplication of epithelial cells within a mesenchymal cellular environment.

## Background

Cell lines that can be propagated indefinitely in culture are a helpful resource with which to study biological processes in cancer biology and experimental therapy. A large number of human cancer cell lines has been generated in the past few decades, and vast scientific information has been gathered from experimental exploitation of these cell lines; however, expanding literature suggests that predictions made based on the behavior of cancer cell lines in vitro, or xenografted into immunosuppressed mice, do not always materialize in clinical applications. In the case of ovarian cancer, this occurrence is highly remarkable. Over 100 human epithelial ovarian cancer (EOC) cell lines have been characterized [1–3] in the past few decades, facilitating the discovery of many pathobiological mechanisms and allowing preclinical testing of novel anti-cancer drugs. Nevertheless, the standard of care for this disease has not changed since the introduction of the platinum/taxane drug combination in the mid-1990s [4]. This leads us to reflect that the experimental models we are using to study cancer in general and ovarian cancer in particular, while useful, are not entirely comprehensive.

A common practice in generating EOC cell lines from primary cultures has been to use a reductionist approach. Within this scope, efforts have always been made to separate the epithelial from the non-epithelial cellular component of the samples, more often than not considered cellular “contaminants” since the role of the tumor environment had yet to be uncovered. Thus, the first cellular components to be separated from the epithelial cells were blood cells, fibroblasts, mesenchymal, and mesothelial cells [5]. The purification of EOC cells has been typically achieved utilizing strategies that take advantage of the differential adhesion and proliferation capacities of the epithelial cells against the non-epithelial cells, leading to the generation of a highly homogenous population of EOC cells that adapt to grow in culture indefinitely [5].

We now know more about the relevance of the interaction between the epithelial component of the tumor and the surrounding stroma in terms of cancer survival, progression, and metastasis; hence, it is important to study the behavior of cancer cells when accompanied by tumor-associated, non-epithelial stromal cells. Herein we provide proof-of-principle for the successful long-term primary culture established from a tumor biopsy of a patient diagnosed with stage IIIc EOC, without purifying any cellular entity in particular. We observed that the complexity of the cellular components of the tumor is what allows the epithelial primary culture phenotype to succeed in growing for a substantial period of time in vitro. We also present evidence suggesting that cells within the epithelial compartment are in an epithelial–mesenchymal hybrid stage, indicating that cells within the tumor biopsy retain phenotypic heterogeneity and plasticity in vitro.

## Methods

### Surgical specimen

A 50-year-old patient had a preoperative diagnosis of an ovarian mass. The patient underwent total abdominal hysterectomy, bilateral salpingo-oophorectomy, and omentectomy. A 20-cm left ovarian mass revealed a centrally solid, yellow-white, papillary structure. The postoperative diagnosis was a stage IIIc optimally debulked, clear cell ovarian carcinoma (O-CCC). The patient received standard IP/IV chemotherapy, but relapsed 13 months after initial surgery and succumbed to the disease 6 months thereafter. The use of the surgical specimen for research purposes was approved by the Institutional Review Board (2007-138) and informed consent from the patient.

### Primary cell culture

A sample from the main ovarian tumor mass was placed in saline in the operating room, sat on ice to maintain viability, transported to the tissue culture room, and handled in a sterile environment. The tissue biopsy was divided into two. One part was fixed in 4 % paraformaldehyde (PFA) and embedded in paraffin; serial paraffin sections (thickness, 5 μm) were obtained and stained with hematoxylin. The second sample of tumoral tissue was enzymatically digested with collagenase Type II. Without further purification, the bulk of the cells was allowed to attach to regular tissue culture flasks and was maintained in RPMI-1640/Ham’s F12 1:1 media supplemented with 10 % fetal bovine serum and antibiotics.

### Cytospin preparations

A 5-day-old primary culture was trypsinized. Suspended cells were fixed at room temperature (RT) for 30 min with 4 % PFA. Finally, approximately 50,000 cells were immobilized in glass slides by cytospin, dried, and subjected to hematoxylin staining or immunostaining for hepatocyte nuclear factor (HNF) 1β.

### Bromodeoxyuridine labeling

Cells isolated from the primary biopsy of O-CCC, after residing in culture for 2 months, were trypsinized and seeded in chamber slides for 11 days. Chamber slides containing cells were exposed to 10 μM 5-bromo-2′-deoxyuridine (BrdU; Molecular Probes, #B23151, Grand Island, NY, USA) for 7 days. At the end of incubation, the cells were fixed with 4 % PFA for 30 min at RT. BrdU incorporated into the DNA was visualized by immunocytochemistry as previously described [6].

### Immunocytochemistry

Cells isolated from the primary biopsy, after spending 2 months in culture, were trypsinized, counted, and allowed to grow for 11 days into chamber slides. Slides containing either cultured cells or cytocentrifuged cells were washed in PBS, fixed with 4 % PFA, and subjected to immunostaining for E-cadherin, vimentin, CD133, BrdU, or HNF1β. Permeabilization and reduction of the non-specific binding was achieved by incubating the cells with 2.5 % normal horse serum, 0.5 % Triton-100 for 20 min at RT. Cells were incubated with the specific primary antibodies in a moist incubation chamber for either 1 h at RT or overnight at 4 °C. Endogenous peroxidase activity was blocked with 3 % H_2_O_2_ for 20 min at RT following incubation with the primary antibody. Antibodies and dilutions were as follows: E-cadherin (#RB-9214-P; 1:30) and vimentin (#RB-9063; 1:2500) (Labvision Corporation, Freemont, CA, USA) for 30 min at RT. BrdU (#A-21303; 1 μg/ml; Molecular Probes) overnight at 4 °C. CD133 (#ab19898; 0.4 μg/ml; Abcam, Cambridge, MA, USA) and HNF1β (#HPA002083; 1:50; Sigma Chemical Company, St. Louis, MO, USA) for 60 min at RT. For immunohistochemistry done in formalin-fixed, paraffin-embedded tissues, antigen retrieval to detect HNF1β expression was carried out by placing tissue sections in a solution of 1 mM EDTA/0.05 % Tween-20, pH 8.0, for 30 min at 96–98 °C, followed by cool-down at RT for 20 min. Samples were incubated with secondary antibody [ImmPRESS HRP Anti-Rabbit (#MP-7401) or Anti-Mouse (#MP-7402) Ig peroxidase; Vector Laboratories, Burlingame, CA, USA] for 30 min at RT. Specific peroxidase activity was developed with the following substrates: ImmPACT DAB Peroxidase (#SK-4105) or ImmPACT NovaRED Peroxidase (#SK-4805) (Vector Laboratories). In negative controls the primary antibody was replaced with 2.5 % normal horse serum (Vector Laboratories).

### Culture of SKOV-3 cells and development of SKOV-3 xenografts

The origin and culture conditions of the SKOV-3 cell line, as well as the establishment of SKOV-3 xenografts in immunosuppressed mice, were previously published in detail [6].

## Results

### Tumor histology

**Fig. 1:**
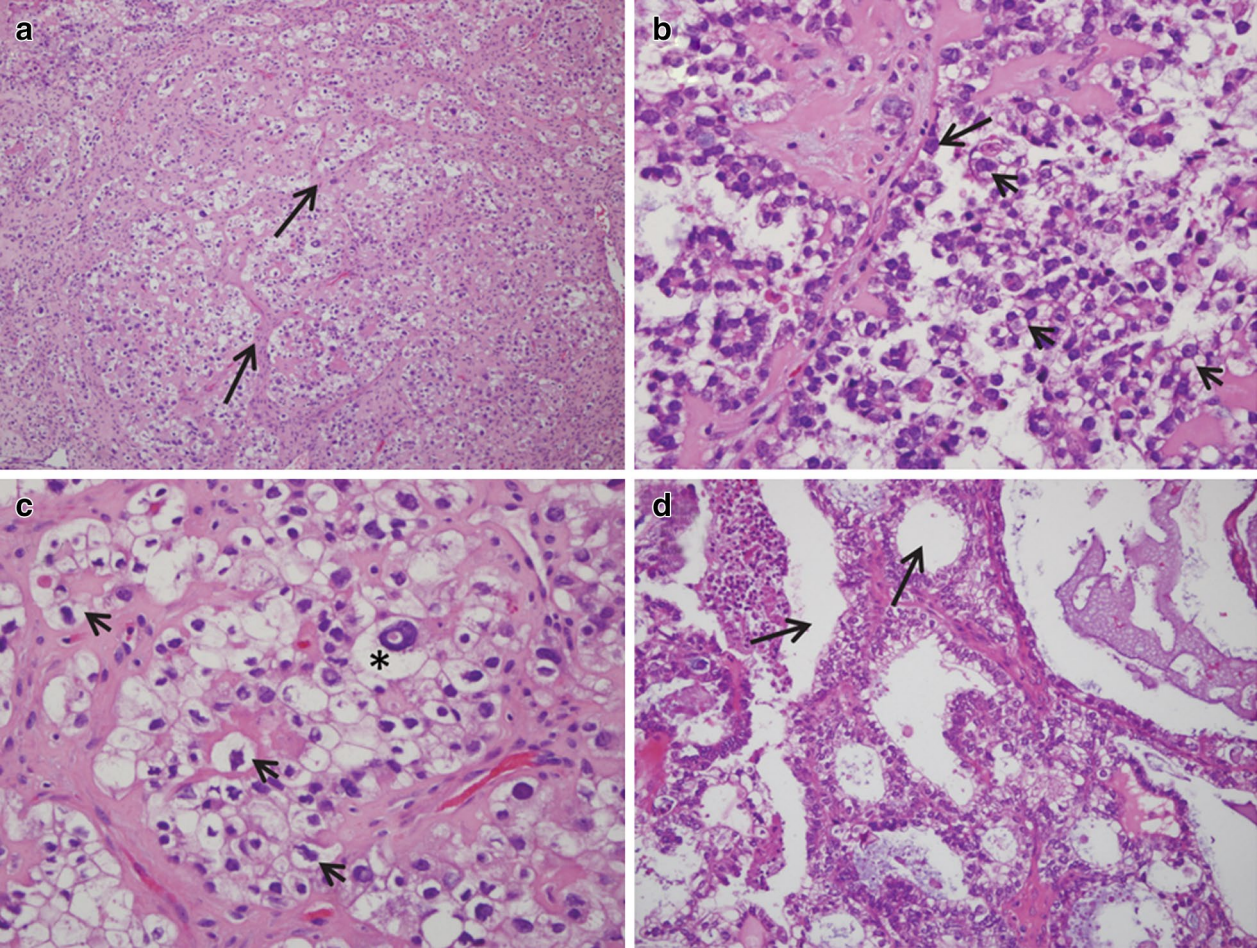
Frozen sections of an O-CCC stained with H&E as diagnosed in the laboratory of pathology. **a** A field (original magnification ×10) containing abundant cells with clear cytoplasm, which are arranged around trabecular connective tissue (*arrows*). **b** (original magnification ×20) and **c** (original magnification ×40) denote the pathognomonic features of O-CCC, with hobnail cells (*arrow* in **b**), and large irregular cells with clear cytoplasm and eccentric prominent nuclei (*arrowheads* in **b**, **c**). The *asterisk* in **c** denotes a cell with clear cytoplasm, and excentric nucleus with a pseudo-inclusion. **d** An image of the tubulocystic aspect (*arrows*) of the clear carcinoma found in the omentum of the same patient

**Fig. 2:**
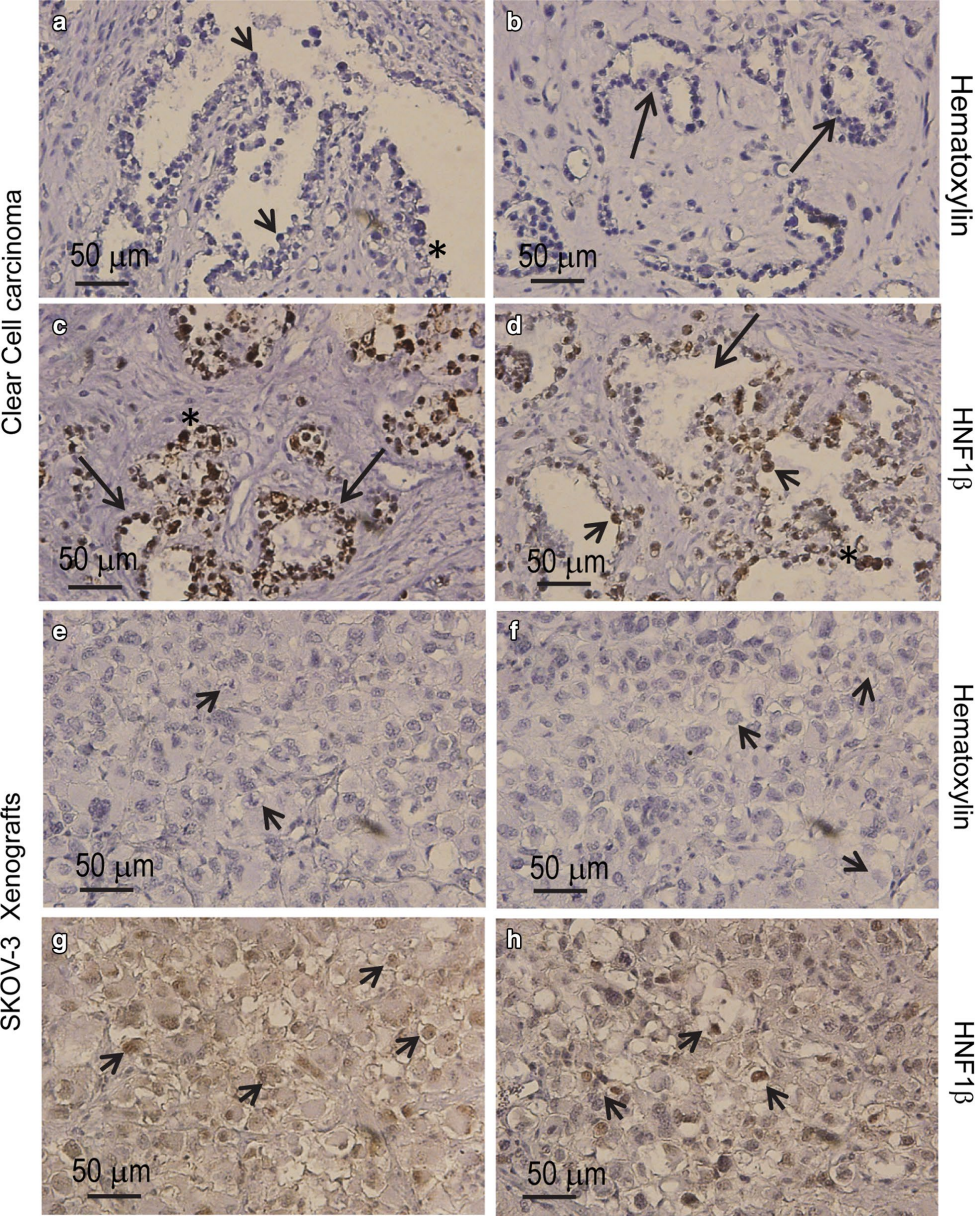
**a**, **b** Representative hematoxylin-stained, paraffin-embedded sections of the tumor biopsy utilized for isolating carcinoma cells for primary culture. Images depict a tubulopapillary pattern (*arrows* in **b**) with hobnail cells (*arrowheads* in **a**) and abundant cells with clear cytoplasm (*asterisk* in **a**). Positive immunohistochemical staining for HNF1β with hematoxylin counterstaining (**c**, **d**) in the biopsy are shown (*arrows*, tubuli with positive nuclei; *arrowheads* depicts positive nuclei in hobnail cells). Hematoxylin-stained sections (**e**, **f**), and HNF1β expression (**g**, **h**) in a xenograft of human SKOV-3 cells developed in immunosuppressed mice (*arrowheads* clear cells)

The histopathological assessment of the sections, performed on various samples representing the ovarian mass, reported an ovarian clear cell carcinoma (O-CCC). Hematoxylin- and eosin-stained (H&E) sections of the tumor mass, as processed in the laboratory of pathology, show an overall clear cell phenotype, with dominant cells having clear cytoplasm and growing in a solid, tubular or papillary pattern, with hobnail cells lining tubules and cysts in a hyalinized stroma. Most epithelial cells are cuboidal and have eccentric and hyperchromatic pleomorphic nuclei, some containing pseudo-inclusions (Fig. 1a–c). A sample from an omental metastasis confirmed the papillary and tubulocystic pattern of the tumor with abundant cells of clear cytoplasm (Fig. 1d). Representative hematoxylin-stained sections of the tumor biopsy utilized for primary culture confirmed the diagnosis of O-CCC, characterized by tubulocystic architecture of the tissue with epithelial cells having little stratification, hobnail cells, and cells with abundant clear cytoplasm (Fig. 2a, b). Positive immuno-detection of a biomarker of O-CCC, HNF1β [7, 8], added to the histopathological diagnosis of the specimen (Fig. 2c, d). HNF1β positivity was also observed in a mouse xenograft developed from the human cell line SKOV-3 (Fig. 2g, h), which depicts the solid morphological features of O-CCC with eccentric nuclei (Fig. 2e–h).

### Primary cell culture findings

**Fig. 3:**
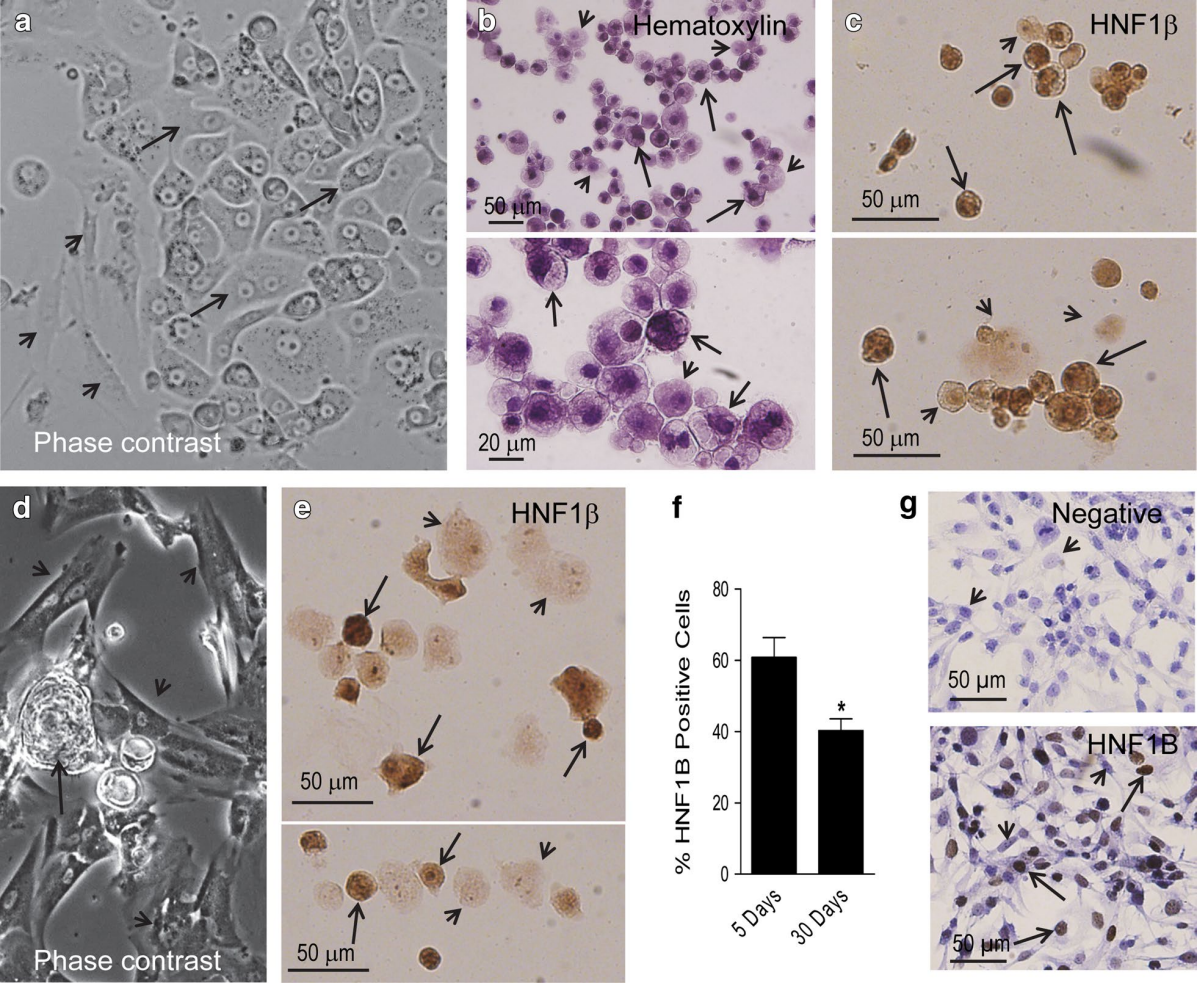
**a** Phase contrast image of a 5-day-old primary culture denoting a monolayer with an epithelial compartment depicting polygonal cells in a pavement-like arrangement (*arrows*) and a mesenchymal compartment (*arrowheads*) (original magnification ×20). **b** Cytospin cell preparations stained with hematoxylin and generated upon trypsinization of a section of a primary cell culture. The fields show two apparent types of cells of heterogeneous sizes, an abundant population of cells depicting dense chromatin and clear cytoplasm (*arrows*), and another population with less dense nuclei with prominent nucleolus, and less clear cytoplasm (*arrowheads*). **c** Cytospin cell preparations subjected to immunocytochemical staining for HNF1β. Many but not all cells show dark positive nuclei; *arrows* show positive-expressing cells whereas arrowheads show cells with negative expression; the panel also shows cells of different sizes. **d** Phase contrast image of a 30-day-old primary culture denoting higher proportion of cells with mesenchymal morphology (*arrowheads*)—when compared to a 5-day culture—always accompanied by epithelial-like cells growing in their vicinity (*arrow*) (original magnification ×10). **e** HNF1β staining in cytospin preparations of a 30-day-old primary culture displaying heterogeneity of expression, positive (*arrows*) or negative (*arrowhead*) cells. **f** Quantitation of the percentage of cells staining positive for HNF1β in cytospin preparations from 5-day or 30-day primary cultures of O-CCC. *p < 0.05 vs. 5 days (Student’s *t*-test). **g** Expression of HNF1β in a culture of SKOV-3 human cancer cells as counterstained with hematoxylin (*upper panel* negative; *lower panel* positive). *Arrows* depict positive immunostaining, whereas *arrowheads* indicate negative immunostaining

Upon enzymatic cellular dispersion of the solid tumor, and after 5 days in culture, the cells that adhered to the plate formed colonies of epithelial appearance, but showed in their surroundings cells with mesenchymal phenotype (Fig. 3a). We considered the mesenchymal cells neighboring the cells with epithelial phenotype a representation of a cellular network preexisting within the in vivo environment; hence, we did not intend to separate them—a method that has been traditionally used to generate epithelial cancer cell lines. Cytospin preparations from a suspension of trypsinized cells, after being cultured for 5 days, revealed the presence of cells of different sizes, most of which displayed a clear cytoplasm and eccentric hyperchromatic nuclei (Fig. 3b). We then assessed the expression of HNF1β in cells from cytospin preparations of a 5-day old culture. About 60 % of the cultured cells displayed positive nuclear HNF1β staining (Fig. 3c, and left column in Fig. 3f). As an in vitro control for HNF1β staining, we utilized a culture of SKOV-3 cells, which shows positive nuclear labelling in the majority of the cells (Fig. 3g). After 30 days of incubation, a primary culture showed an apparent increase in the proportion of cells having a mesenchymal morphology when compared to a 5-day primary culture (Fig. 3d). These changes were associated with a significant decline in the percentage of cells that labeled positive for the epithelial O-CCC maker HNF1β (Fig. 3e, and right column in Fig. 3f).

**Fig. 4:**
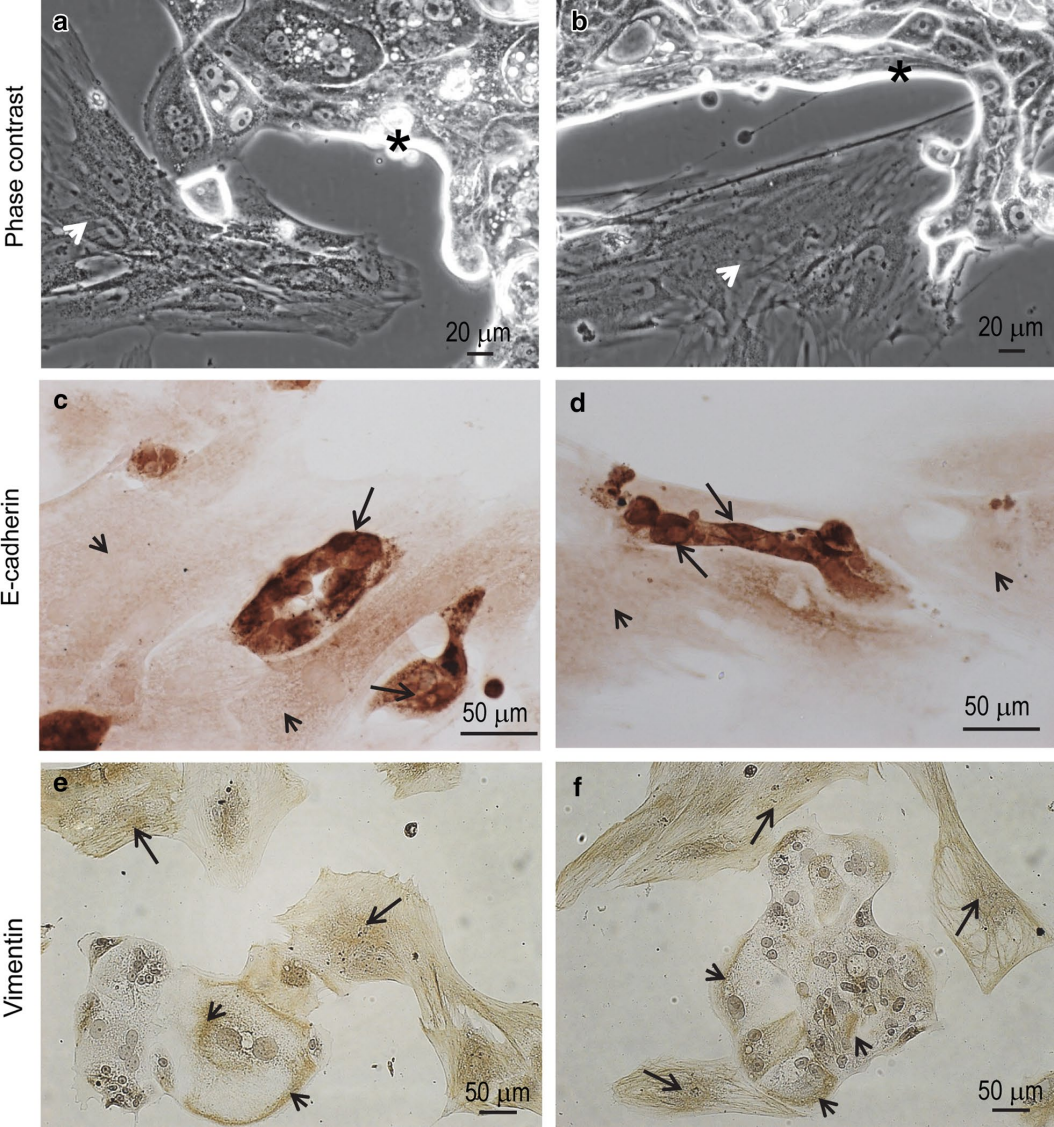
**a**, **b** Phase contrast images depicting the coexistence of epithelial (*asterisks*) and mesenchymal (*arrowheads*) cellular compartments after 8 weeks in culture. **c**, **d** E-cadherin immunoreactivity is shown in epithelial cells (*arrows*) but not in mesenchymal cells (*arrowheads*). **e**, **f** Vimentin immunostaining is positive mainly in the mesenchymal cells (*arrows*), yet some positivity is also observed in the cytoplasm of epithelial-like cells (*arrowheads*)

After 60 days of incubation, and with periodic media changes, the culture still displayed a combination of epithelial foci surrounded by mesenchymal-like cells (Fig. 4a, b). When we compared the phase contrast micrographs taken 5 days after initial plating versus those taken 2 months later (Figs. 3a vs. 4a, b), the net growth of the epithelial component of the culture was evident; yet, mesenchymal cells seemed to increase in relative proportion, as well, accompanying and surrounding the epithelial compartment.

After 2 months in culture, when we sub-cultured the cells shown in Fig. 4a, b in chamber-slides, the cells with epithelial morphology—but not those with mesenchymal morphology—stained positive for the epithelial marker E-cadherin (Fig. 4c, d). The mesenchymal marker vimentin stained positive mostly in the mesenchymal compartment; however, positive staining also appeared in the cytoplasm of some cells of the epithelial compartment (Fig. 4e, f), suggesting the existence of a transitional epithelial-mesenchymal phenotype.

**Fig. 5:**
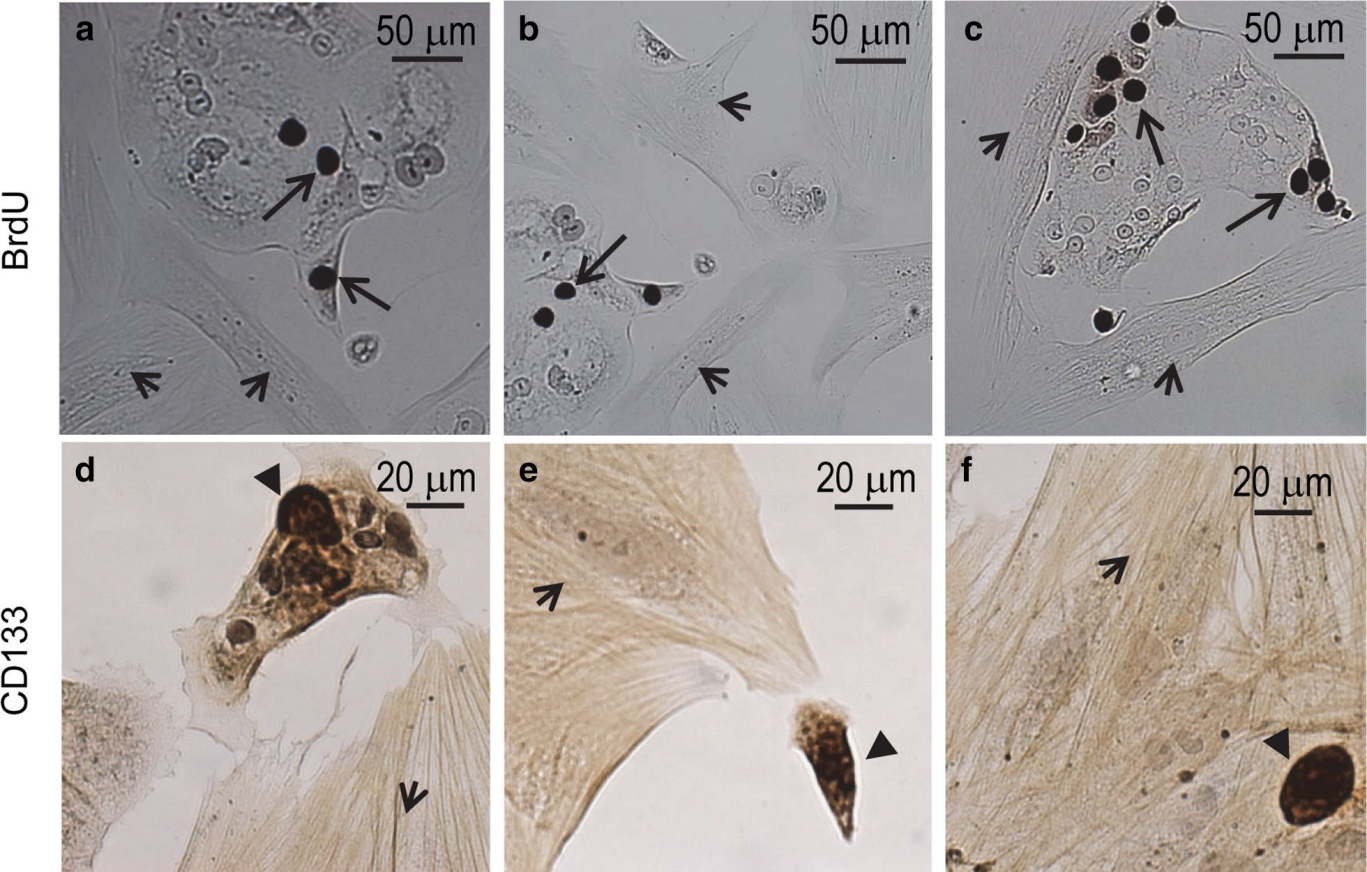
**a**–**c** Bromodeoxyuridine (BrdU) was incorporated into cells of the epithelial compartment (*arrows*) but not into cells of the mesenchymal compartment (*arrowheads*). **d**–**f** CD133 positive cells (*black triangles*) associated to both the epithelial (**d**) and the mesenchymal (*arrowheads*) compartments (**e**, **f**)

The synthesis of DNA within the epithelial compartment, but not the mesenchymal compartment, was confirmed by BrdU incorporation (Fig. 5a–c). Finally, after 2 months of incubation, the heterogeneous culture retained cells that stained positive for the stem cell marker CD133, described by many as being expressed in ovarian tumor-initiating cells [9–12]. CD133 expression was observed in the plasma membrane of few cells, which were associated with both the epithelial and the mesenchymal compartments of the cell culture (Fig. 5d–f).

## Discussion

Most EOC cell lines available for research purposes have been developed from cancer cells found within ascites and pleural effusions of patients diagnosed with EOC. A limited number of EOC cell lines were developed from solid primary and metastatic biopsies. In both cases, the main efforts placed into the development of the EOC cell lines were geared at isolating clones with epithelial-only phenotype, with the purpose of selecting the ovarian cancer cells.

During the past 20 years, the critical role of the tumor microenvironment, mainly composed of tumor-associated cells, has been unveiled (reviewed in [11]). It is now clear that the relative low heterogeneity of EOC lines seems insufficient to reflect the inter-cellular complexity found within a tumor. Thus, in the present work, we provide a snapshot of the behavior of a primary cell culture derived from a biopsy, aiming to preserve the main cellular components of the original tumor mass. The bulk of cells released form the biopsy had clear cytoplasm, which is characteristic of a clear cell carcinoma. Furthermore, 60 % of the cells showed nuclear staining for the biomarker HNF1β, a protein considered to be pivotal in the pathogenesis of O-CCC as evolving from endometrioid lesions [8, 13], and found to have high sensitivity and specificity to differentiate O-CCC from high-grade serous ovarian cancers [7].

We provide new evidence that, in addition to the epithelial phenotype denoted by a pavement-like arrangement of the cells, there was a second phenotype with mesenchymal features composed of vimentin-positive, E-cadherin negative cells always located in close proximity to the epithelial edge. This suggests that a cooperative interaction might exist between these two cellular compartments with the likelihood for the mesenchymal cells to molecularly prompt the epithelial cells to undergo proliferation followed by transition to, again, a mesenchymal phenotype (i.e. EMT). These observations are in agreement with a recent study in which two well-known EOC lines, when co-cultured with primary mesenchymal stem cells, reprogrammed their transcriptome leading to the acquisition of traits that promote migration, invasion, proliferation, and resistance to platinum [14]. Significantly enough in this last study, the mesenchymal cells were able to sustain proliferation of EOC in vitro in the absence of serum and cytokines, unveiling the involvement of cells from the microenvironment that are likely hijacked by the tumor cells to promote their own persistence via paracrine mediators.

Another study suggests that resident fibroblasts can be reprogrammed into cancer-associated fibroblasts upon epigenetic changes triggered by factors released from the EOC cells. Subsequently, the reprogrammed fibroblasts upregulate the expression of cytokines and chemokines that promote growth and metastasis of the EOC cells [15]. We observed DNA-synthesizing EOC cells in the epithelial compartment but not in the mesenchymal compartment, suggesting that paracrine pro-proliferative factors may be provided by the mesenchymal cells to the EOC cells.

The present study also suggests that there is a mesenchymal–epithelial plasticity in culture based on two key observations. On the one hand, cells with epithelial-like morphology stain for the mesenchymal marker vimentin, denoting that they are in a hybrid, epithelial–mesenchymal stage. Such a hybrid stage was described in human ovarian cancer cells freshly isolated from mouse xenografts, or, in situ, in human biopsies in which cancer cells simultaneously express the epithelial marker EpCAM and the mesenchymal marker vimentin [16]. On the other hand, we observed that the overall proportion of cells expressing the O-CCC epithelial biomarker HNF1β declines with time in culture in association with an apparent expansion of the mesenchymal compartment. This observation suggests the likely existence of a transdifferentiating phenomenon, taking into account that the epithelial but not he mesenchymal cells are the ones that divide in culture. This plasticity may provide an explanation for the retention, within both epithelial and mesenchymal compartments, of cells expressing the O-CCC stem-like marker CD133 [12], which is usually lost when cells undergo lineage-associated differentiation [16].

## Conclusion

This work provides evidence that cells separated in bulk from a biopsy of an O-CCC can be maintained in culture for several months, depicting the coexistence and cooperative interaction among two cellular compartments: one epithelial that retains the O-CCC marker HNF1β and another mesenchymal.

## Abbreviations

O-CCC: clear cell carcinoma of the ovary
HNF1β: hepatocyte nuclear factor 1β
EOC: epithelial ovarian cancer
PFA: paraformaldehyde
RT: room temperature
BrdU: 5-bromo-2′-deoxyuridine
H&E: hematoxylin and eosin
